# Observed Versus Expected Use of Diagnostic Imaging and Radiotherapy in Prostate Cancer in the Lazio Region (Italy): An Integrated Population-Based and Literature-Informed Framework for Healthcare Planning

**DOI:** 10.3390/healthcare14111597

**Published:** 2026-06-05

**Authors:** Margherita Ferranti, Angelo Nardi, Matilde Zijno, Emanuela Carloni, Sara Lopes, Ilaria Cozzi, Enrica Santelli, Sara Farchi, Daniela D’Ippoliti, Paola Michelozzi, Luigi Pinnarelli

**Affiliations:** 1 Department of Epidemiology—Regional Health Service, Local Health Authority Rome 1, Via Cristoforo Colombo, 112, 00147 Rome, Italy; a.nardi@deplazio.it (A.N.); m.zijno@deplazio.it (M.Z.); e.carloni@deplazio.it (E.C.); s.lopes@deplazio.it (S.L.); i.cozzi@deplazio.it (I.C.); e.santelli@deplazio.it (E.S.); d.dippoliti@deplazio.it (D.D.); p.michelozzi@deplazio.it (P.M.); l.pinnarelli@deplazio.it (L.P.); 2 Area Rete Ospedaliera E Specialistica, Direzione Regionale Salute E Integrazione Sociosanitaria Regione Lazio, 00168 Rome, Italy; sfarchi@regione.lazio.it

**Keywords:** prostate cancer, healthcare planning, cancer registry, radiotherapy utilization, diagnostic imaging, care pathways, demand estimation, health services research

## Abstract

**Highlights:**

**What are the main findings?**

- A population-based framework integrating cancer registry data, administrative databases, and literature evidence was developed to estimate diagnostic imaging and radiotherapy needs in prostate cancer.
- In the Lazio Region (central Italy), literature-informed expected utilization of key services consistently exceeded observed utilization, suggesting potential variations relative to literature-informed projections.

**What are the implications of the main findings?**

- Comparing expected and observed service use can support healthcare planning by identifying divergences between literature-based estimates and real-world utilization patterns.
- The proposed framework is reproducible and adaptable to other oncological pathways and healthcare systems to support healthcare planning.

**Abstract:**

**Background/Objectives**: Accurate estimation of healthcare service requirements is essential for planning oncological care pathways, particularly in resource-constrained settings. We developed a combined methodological framework integrating pragmatic rapid literature reviews and population-based cohort analyses to estimate expected and observed utilization of diagnostic imaging and radiotherapy services along the prostate cancer care pathway in the Lazio Region (central Italy). **Methods**: Observed utilization was assessed using incident prostate cases recorded in the Lazio Region Cancer Registry in 2019 (n = 3047) and provisional cases in 2022 (n = 3254), through linkage with regional health information systems. For each cohort, 16 indicators (magnetic resonance imaging, biopsy, metastatic staging imaging, radiotherapy) were calculated, estimating usage proportions, median delivery times, and stratifying by age class. The expected requirement was estimated through four rapid literature reviews of the Italian literature and applied to incident prostate cancer cases. **Results**: Sixteen indicators covering diagnostic, staging, and therapeutic services were analyzed. Observed utilization varied across the prostate cancer care pathway, including diagnostic imaging, biopsy, surgery, and radiotherapy. For example, radiotherapy was expected in approximately 33–42% of patients, whereas the observed utilization was 23.00% of cases (95% CI 21.48–24.61) in 2019 and 22.52% of cases (95% CI 21.07–24.03) in 2022. The provision of imaging for metastatic staging—40.85% (95% CI 39.06–42.67) in 2019 and 34.84% (95% CI 33.19–36.53) in 2022—was lower than the expected value of 50%. Differences in utilization patterns by age group and timing of service delivery were observed. Literature-informed expected estimates exceeded observed utilization for several services, including both diagnostic and therapeutic components of the pathway. **Conclusions**: Overall, variations were identified between observed utilization and literature-informed expected estimates, suggesting the utility of evaluating potential areas for healthcare pathway optimization. This reproducible and adaptable methodology can be applied to other care pathways and healthcare settings, thereby supporting strategic resource allocation and continuous monitoring.

## 1. Introduction

The assessment of healthcare service requirements represents a major public health challenge, particularly in contexts characterized by constrained resources and growing population health needs. Such analyses are essential to inform healthcare planning and to ensure alignment between evidence-based clinical guidelines and their implementation within specific organizational frameworks. However, in the literature there is a lack of methodological approaches used to estimate the needs of the entire care process of a specific disease. Indeed, existing research has primarily focused on estimating requirements for the healthcare workforce rather than for healthcare services themselves, with the aim of quantifying total resource needs in terms of physicians and nurses or concentrating on single components of care—such as radiotherapy—to estimate changes or trends within a defined geographical area [1,2,3]. Therefore, as the choice of the method depends on the specific purpose and setting of the analysis, there is a need for more general, integrated and pragmatic approaches, suitable for different clinical contexts and not focused on a specific aspect [4,5]. In particular, the integration of different approaches could be more useful for the implementation of clinical pathways, including those developed for complex and heterogeneous diseases, such as prostate cancer.

Within this broader context, integrated care pathways for prostate cancer represent a paradigmatic example. Prostate cancer is among the most common malignancies in men worldwide and is characterized by heterogeneous clinical trajectories. In Italy, recent estimates showed an annual incidence of prostate cancer of about 39,000 new diagnoses, with the Lazio Region showing the third highest absolute level [6,7]. Heterogeneity is shown in disease presentation, as it could be asymptomatic or not, in clinical significance, as it could range from indolent to very aggressive, and in treatment choice, which could depend also on individual factors such as age. As complexity arises from the whole clinical pathway, studies aimed at implementing clinical pathways should simultaneously try to take into account all these different aspects.

In the Lazio Region (central Italy), within the framework of the Regional Oncological Network, a new clinical pathway for patients with suspected prostate cancer has been developed. Designed to cover the full continuum of care—from diagnosis to treatment and follow-up—and aligned with current clinical guidelines, the pathway specifies the appropriate diagnostic examinations and therapeutic interventions for each disease stage at diagnosis. Before its implementation, estimating the required volume of diagnostic imaging procedures, including magnetic resonance imaging (MRI), scintigraphy, computed tomography, as well as radiotherapy (RT) services, is essential to ensure adequate resource allocation.

To achieve this aim, based on existing models in the literature, two main approaches to assessing healthcare service requirements can be distinguished: utilization-based and need-based. Utilization or demand-based methodologies rely on current patterns of health service use, as observed in healthcare data sources. It reflects actual service delivery within a specific healthcare system and may be influenced by organizational constraints, access barriers, and existing capacity. In contrast, need-based approaches—typically informed by clinical guidelines and literature evidence—aim to estimate the level of care that should be provided under optimal conditions on the basis of epidemiological expectations, such as disease incidence and stage distribution. Integrating these two perspectives offers a powerful framework for identifying the gap between delivered care and underlying clinical needs and supporting data-informed healthcare planning.

Within this context, the Department of Epidemiology of Lazio Region Health Service was mandated to estimate resource requirements for the new prostate cancer clinical pathway, to support improved resource allocation across the Local Health Authorities of the Region. Therefore, the study aims to:

(1) Provide a pragmatic estimation of expected needs based on evidence from the scientific literature and recommendations from clinical guidelines, with a specific application to the regional context;
(2) Analyze observed utilization through the use of available population-based and administrative data;
(3) Compare the gap between observed and expected utilization of healthcare services.

## 2. Materials and Methods

This study was conceived as a combined methodological framework integrating two complementary components: literature-informed estimation of expected healthcare service demand through pragmatic rapid reviews, and population-based analysis of observed healthcare service utilization using cancer registry and administrative healthcare data. The integration of these components enabled a comparative assessment between expected and observed utilization of diagnostic imaging and radiotherapy services within the prostate cancer care pathway.

### 2.1. Pragmatic Rapid Reviews

To estimate population needs in terms of diagnostic procedures and radiotherapy, we conducted four pragmatic rapid reviews to assess the expected demand in the Italian context based on the available literature. Rapid reviews are commonly used to synthesize evidence within a specific field and context in order to inform policymakers within a short time frame [8]. The use of literature-based estimates to derive expected utilization was motivated by the need to approximate clinical demand independently of local healthcare system constraints. While the regional administrative and registry data provide a robust basis for measuring observed utilization, they inherently reflect current practice patterns and resource availability, and therefore cannot be used alone to estimate unmet need. Literature-derived parameters, based on clinical studies and guideline-informed populations, were used to approximate expected service utilization under conditions of appropriate care.

In particular, these reviews were intentionally conducted as pragmatic rapid reviews, prioritizing timeliness and contextual relevance over methodological completeness typically required in full systematic reviews. Accordingly, four authors performed the reviews. Two authors independently conducted the screening for two of the rapid reviews, while another two authors screened the remaining two reviews. In cases of disagreement or uncertainty, final decisions were made by consensus among all the authors involved in the rapid reviews. No formal risk of bias assessment, study weighting procedures, or meta-analytic synthesis methods were applied. Instead, summary estimates were derived using median values and reporting ranges (minimum–maximum) and interquartile ranges (IQRs), when available. In addition, sensitivity analyses were conducted using alternative parameter values based on the lower and upper bounds observed in the literature. This methodology reflects the variability of the available evidence and provides robust and transparent approximations of expected parameters for healthcare planning purposes [8].

This approach was considered appropriate given the heterogeneity of study designs, populations, and reported outcomes, as well as the primary aim of informing service planning rather than producing pooled effect estimates [8].

Specifically, we aimed to estimate: (1) the proportion of patients with MRI performed for suspected prostate cancer who subsequently underwent prostate biopsy; (2) the proportion of biopsies resulting in a diagnosis of prostate cancer; (3) the distribution of newly diagnosed prostate cancer cases according to D’Amico risk categories (low, intermediate, and high); and (4) the proportion of newly diagnosed patients who underwent radiotherapy. The D’Amico risk classification was selected because it provides the criteria adopted by the Lazio Region clinical pathway to identify patients eligible for further diagnostic imaging procedures (e.g., whole-body CT). Sensitivity analyses were also conducted using alternative risk classification systems.

For each objective, a pragmatic rapid review was conducted using MEDLINE (via PubMed). An approach based on the population, intervention, comparison and outcome (PICO) method was used, and four different search strings were developed (See Supplementary Table S1). Studies were considered eligible if they were conducted in Italy and included either individuals with suspected prostate cancer (for estimates related to MRI and biopsy outcomes) or patients with a diagnosis of prostate cancer (for estimates related to D’Amico risk categories and radiotherapy use). Articles were included regardless of their primary study objective, considering sufficient that the outcome of interest was reported or could be calculated. The expected-demand model was constructed by applying D’Amico risk class proportions—derived from the pragmatic rapid literature reviews—to the total incident population. We included only studies based on Italian data for both practical and methodological reasons: first, extending the analysis to other countries would have led to the inclusion of a large number of articles not suitable for the purpose of a rapid review; on the other hand, consistent with the study aims, the decision to focus on national data was intended to improve the comparability of estimates, due to similarities in healthcare access, healthcare system organization, and individual characteristics. Indeed, the Lazio Region is a significant part of the Italian population. This means that it represents the Italian population across various demographic, health, and lifestyle indicators, confirming that the regional distribution is broadly consistent with national patterns https://osservatoriosullasalute.it/rapporto-osservasalute (accessed on 12 May 2026).

To obtain estimates relevant to current practice, we limited inclusion to studies published between 1 January 2016 and 28 February 2026. Studies were excluded if they focused on excessively narrow subpopulations of patients with suspected prostate cancer (e.g., only individuals with a single MRI lesion or with two or more lesions; only patients with positive or negative digital rectal examination), as such restrictions would limit the generalizability of the estimates to the broader population.

Data were extracted using a standardized form capturing bibliographic information and outcomes of interest. To estimate expected demand, medians and interquartile ranges (IQRs) were calculated for each parameter. When too few studies were available, individual study estimates were reported.

### 2.2. Data Sources

Prostate cancer incidence data for the Lazio Region were obtained from the Lazio Region Cancer Registry (LRCR), a population-based registry that identifies incident cancer cases through systematic individual-level record linkage across multiple Healthcare Information System (HIS) databases, as previously described in detail [9]. Briefly, the LRCR integrates the traditional data sources commonly used by cancer registries, including Hospital Discharge Records (HDRs), pathology reports, death certificates, and ancillary datasets. All residents of the Lazio Region are assigned a unique encrypted personal identifier, which enables accurate linkage across data sources and prevents double counting. The LRCR manages approximately 35,000 new cancer cases each year and produces both provisional incidence estimates based on up-to-date administrative HIS data and fully consolidated incidence data validated by trained registrars; consolidated data for all cancer sites are currently available up to 2019.

For the purposes of this study, we used consolidated prostate cancer incidence data for 2019 and for 2022 (the most recent year available) the “provisional cohort” identified through a validated algorithm of administrative flows. All prostate cancer cases (ICD-O-3 topography code C61) were extracted from the LRCR database. The LRCR operates according to standardized international protocols for population-based registries. As part of its routine data consolidation process, the LRCR ensures geographical and clinical accuracy by excluding non-residents through systematic cross-referencing with the regional Healthcare Assistance Database. Similarly, to isolate incident cases from prevalent or recurrent disease, the LRCR employs a multi-source record linkage system supported by a permanent look-back period dating back to 2001. Individual-level record linkage was performed to integrate information across data sources including the Outpatient Specialist Care Information System (OSCIS) and HDRs. Information on vital status was obtained from HDRs, the Health Emergency Information System, Healthcare Assistance Registry and death certificates (available only for the 2019 cohort).

#### Cohort Comparability

A methodological distinction exists between the two study cohorts in terms of data maturity and ascertainment sources. As previously described, the 2019 cohort is derived from a consolidated population-based cancer registry, with complete histological and clinical validation. On the other hand, the 2022 cohort representing the latest available data year, constitutes the provisional incident population for the LRCR and lacks the systematic record linkage with pathology reports and mortality data available for 2019. This limitation was not considered a major concern for the prostate cancer cohort given the high sensitivity of HDRs for this malignancy.

### 2.3. Study Design and Population

The population-based cohort component of the framework was conducted to analyze the observed utilization of healthcare services among patients with incident prostate cancer. Sixteen indicators were defined for each cohort to capture the utilization of selected diagnostic and therapeutic services. All patients with an incident diagnosis of prostate cancer recorded in the LRCR in 2019 or 2022 were included. The date of cancer diagnosis was defined as the index date. Patients were followed to assess the utilization of healthcare services relevant to prostate cancer management. Information on diagnostic and therapeutic healthcare services was obtained through record linkage with the HDRs and the OSCIS.

### 2.4. Outcomes

The study evaluated the observed utilization of diagnostic and therapeutic services, including prostate MRI, biopsy, surgical treatment, imaging for metastatic staging (scintigraphy, whole-body CT, PSMA PET/CT) and radiotherapy. Radiotherapy was analyzed in three ways: any radiotherapy, adjuvant radiotherapy (following surgery), and radiotherapy delivered in the absence of surgical intervention. HIS did not allow a more granular characterization of radiotherapy, preventing distinction between specific treatment modalities. For each cohort, a set of indicators was calculated (See Supplementary Table S2). For each service, the proportion of patients receiving the procedure and the median time from diagnosis—or surgery, when applicable—to service delivery were estimated. To ensure clinical relevance, healthcare services were identified by linking the incident cohorts to regional administrative databases using a 12-month observation window around the index and/or surgery date. Within this period, specific time windows were defined to capture service utilization related to the initial pathway: for instance, diagnostic MRI and biopsy were assessed within 60 and 180 days prior to diagnosis, while surgical and radiation treatments were identified within 365 days following the index date. A summary of the indicators and their corresponding observation windows is presented in Supplementary Materials in Table S3.

### 2.5. Statistical Analysis

Descriptive analyses were performed to evaluate the observed utilization for each healthcare service. Results were reported as proportions and median times to service utilization. Ninety-five percent Confidence Intervals (95% CI) were calculated using the Wilson score method. Analyses were conducted separately for the 2019 and 2022 cohorts and stratified by age group where appropriate.

A one-year mortality follow-up was conducted for all cases, censoring for all-cause mortality during the observation window. Specifically, patients who died before the end of an indicator-specific time window were excluded from the corresponding analysis to avoid underestimating service utilization.

Statistical analyses were performed using SAS Enterprise Guide software, Version [8.3]. Copyright © (SAS Institute Inc. SAS and all other SAS Institute Inc. product or service names are registered trademarks or trademarks of SAS Institute Inc., Cary, NC, USA), and R software version 4.5.3 (R: A language and environment for statistical computing. R Foundation for Statistical Computing, Vienna, Austria. URL: https://www.R-project.org/).

## 3. Results

### 3.1. Literature-Based Benchmark Generation

Flow diagrams for each pragmatic rapid review are provided in the Supplementary Materials Figures S1–S4. The literature searches identified 422, 216, 171, and 532 records for the reviews, estimating the proportions related to MRI findings, prostate biopsy outcomes, D’Amico risk categories, and radiotherapy use, respectively. Following the screening process, 6, 35, 7, and 3 studies were included for the respective reviews. In addition, some studies retrieved for one review were also included in another review when relevant to a different objective.

Extracted estimates from the included studies are reported in Tables S4–S7. For PIRADS score, percentage of positive biopsies and D’Amico score, results were summarized in Table S8. Overall, substantial between-study variability was observed, particularly for the proportion of patients with lesions detected on MRI.

The median proportion of positive MRI findings (defined as a PIRADS score ≥ 3, generally considered an indication for biopsy) was 70.0%, with an IQR of 52.5–76.6%. The proportion of MRI examinations with PIRADS ≥ 3 lesions showed marked heterogeneity, ranging from approximately 37.5% to 89.5% across studies.

The median proportion of positive biopsies was 60.4% (IQR 52.6–64.8%) for prostate cancer overall and 39.0% (IQR 34.8–48.5%) for clinically significant prostate cancer. Substantial variability was observed in these outcomes, with prostate cancer detection rates ranging from approximately 43% to 78.8%, and clinically significant prostate cancer ranging from 24% to 62.7%.

Regarding risk stratification, the median distribution according to D’Amico classification was approximately 24.0% (IQR 22.6–33.2%) for low-risk, 49.5% (IQR 45.1–52.8%) for intermediate-risk, and 24.1% (IQR 17.1–26.2%) for high-risk disease. The distribution of D’Amico risk categories also showed variability, with low-risk cases ranging from 21% to 48%, intermediate-risk from 38% to 62.3%, and high-risk from 14% to 32.6%. Only one study further distinguished intermediate-risk patients, reporting 16.0% with favorable and 30.4% with unfavorable intermediate-risk disease. Sensitivity analyses incorporating alternative risk classification systems yielded minimal differences (See Supplementary Table S6).

Among the three studies reporting radiotherapy use, the proportion of patients undergoing this therapy ranged from 33.3% to 42.4%, with a median value of approximately 40%. Additionally, one of them reported an 11.7% increase in radiotherapy use following radical prostatectomy; this finding is consistent with results from another study not included in the review, which reported a corresponding estimate of 14.8%. Included studies did not allow a more detailed distinction between the different types of radiotherapy performed.

Sensitivity analyses using alternative assumptions based on the minimum and maximum values observed in the literature confirmed the robustness of the main findings. For instance, when considering the lowest reported radiotherapy utilization (33.3%), the expected proportion remained substantially higher than the observed utilization in both cohorts (~22–23%). Similarly, using the upper bound (42.4%) further increased the estimated gap.

### 3.2. Observed Healthcare Utilization

**Figure 1:**
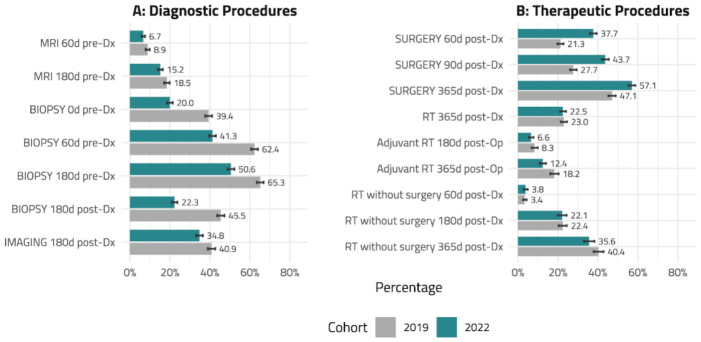
Diagnostic (**A**) and treatment (**B**) pathways for the 2019 and 2022 cohorts. This figure presents the percentage of patients with at least a diagnostic (biopsy, MRI and imaging for metastatic staging) or therapeutic (surgery and RT) procedure within specified time windows (e.g., 60 days pre- or post-diagnosis/surgery) for the 2019 and 2022 cohorts.

A total of 3047 patients were included in the 2019 cohort and 3254 in the 2022 cohort. Table 1 presents the age characteristics of the two cohorts, while Figure 1 shows the percentage of patients with at least a diagnostic (biopsy, MRI and imaging for metastatic staging) or therapeutic (surgery and RT) procedures within specified time windows for the 2019 and 2022 cohorts.

With respect to diagnostic procedures, both cohorts showed similar proportions of patients undergoing MRI within 60 or 180 days before diagnosis. In the 2019 cohort, 65.28% (95% CI 63.57–66.95) of patients underwent biopsy, whereas this proportion was lower in the 2022 cohort. When considering the 180 days after diagnosis, 40.85% (95% CI 39.06–42.67) of patients underwent imaging for metastatic evaluation in the 2019 cohort and 34.84% (95% CI 33.19–36.53) in the 2022 cohort. The median time from diagnosis to metastatic imaging was 40 days for the 2019 cohort and 37 days in the 2022 cohort.

Regarding therapeutic interventions, notable differences emerged between cohorts: 21.28% (95% CI 19.82–22.80) of patients in the 2019 cohort underwent surgery within 60 days of diagnosis, compared with 37.72% (95% CI 36.06–39.42) in the 2022 cohort. Radiotherapy utilization was consistent across cohorts and was higher among patients who did not undergo surgery; the median time from diagnosis to the start of radiotherapy was 198 days in 2019 and 190 days in 2022.

Stratified analyses by age group revealed substantial differences in treatment patterns (see Supplementary Table S9, Figures S5 and S6). Younger patients were more likely to undergo surgery, whereas older patients more frequently received radiotherapy. Patients aged 85 years and older consistently showed lower treatment proportions, indicating more conservative management strategies in the oldest age groups.

**Figure 2:**
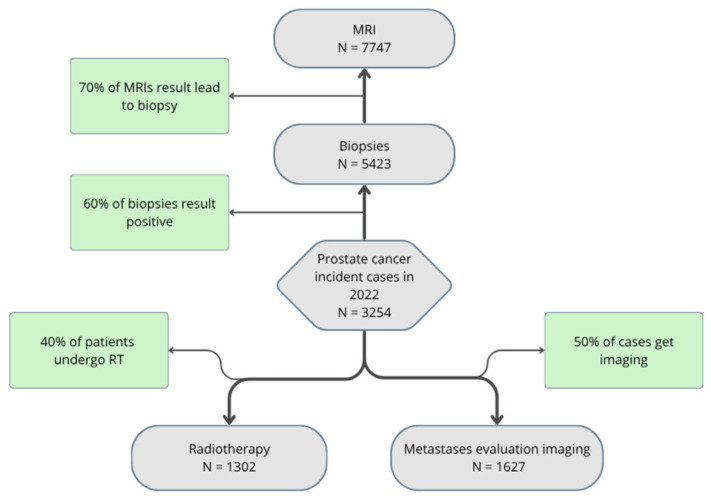
Theoretical Framework and Expected-Demand Model for Prostate Cancer Pathways. The flowchart illustrates the sequential steps in the diagnostic and therapeutic pathway for prostate cancer incident cases in 2022 and provides procedural estimates based on literature estimated benchmarks.

Figure 2 presents estimates of expected healthcare service needs. Based on the literature and considering the 3254 incident cases recorded in the Lazio Region in 2022, the estimated need corresponds to 5423 prostate biopsies and 7747 prostate MRI examinations. Expected pre-diagnostic volumes refer to men investigated for suspected prostate cancer (not only incident cases).

**Figure 3:**
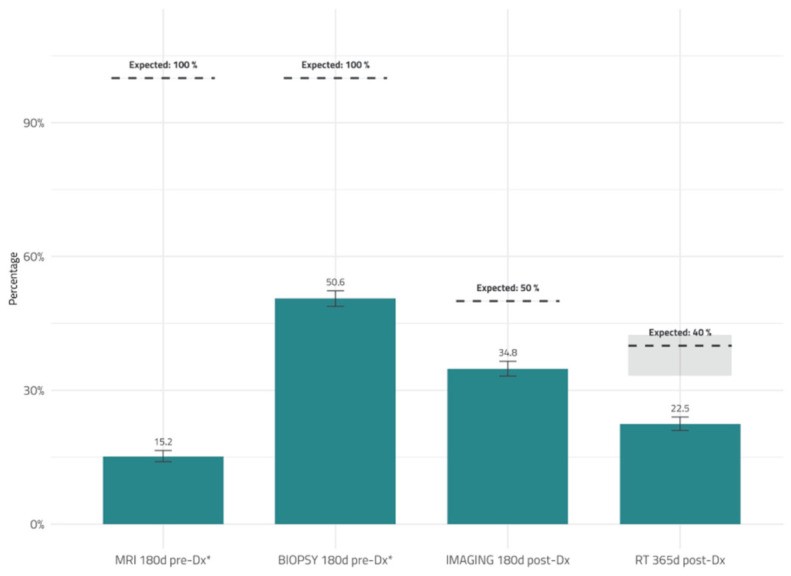
Comparison between expected and observed values. Observed values are based on incident prostate cancer cases in 2022 (the most recent available data). Expected imaging values are based on the D’Amico risk stratification. Expected RT values and ranges are based on pragmatic rapid reviews. * According to guidelines updated through February 2026, all incident cases should undergo MRI and biopsy before receiving a diagnosis of prostate cancer; therefore, the expected value is 100%.

Post-diagnosis, the literature suggests that approximately 40% of patients undergo radiotherapy, corresponding to an estimated need of 1302 radiotherapy treatments. This estimate exceeds observed utilization, as radiotherapy was delivered in 23.00% (95% CI 21.48–24.61) of cases in the 2019 cohort and in 22.52% (95% CI 21.07–24.03) of cases in the 2022 cohort (Figure 1 and Figure 3). For imaging aimed at metastatic staging, reported utilization is approximately 50%, based on the assumption that imaging was indicated in all high-risk patients and in those with unfavorable intermediate-risk disease, who were assumed to represent approximately half of the intermediate-risk population. Although the observed proportions were lower (Figure 1), the estimated need corresponds to 1627 procedures (Figure 3). Overall, observed utilization appeared lower than the corresponding literature-derived expected estimates, particularly radiotherapy and staging imaging (Figure 3).

This analytical approach, based on regional data, can be readily extended to individual Local Health Authorities or Districts by stratifying incident cases according to patients’ place of residence at diagnosis.

## 4. Discussion

This study provides an integrated assessment of observed utilization and expected need for diagnostic and therapeutic services along the prostate cancer care pathway in the Lazio Region. By analyzing incident cohorts from 2019 and 2022, the findings highlight relevant discrepancies between real-world service use and needs estimated on the basis of epidemiological evidence and clinical guideline recommendations. While the utilization of some diagnostic procedures, such as prostate magnetic resonance imaging and radiotherapy, showed overall consistency across cohorts, substantial differences emerged in the timing and uptake of surgical interventions. The expected needs tended to be higher than observed utilization, particularly for radiotherapy and imaging procedures aimed at metastatic staging, suggesting variations between expected and observed utilization that may reflect a combination of organizational factors, evolving clinical practices, and appropriate patient-level decision-making. In particular, age-related differences in treatment patterns highlight the importance of interpreting these findings within a clinical context. Differences between observed and expected utilization should not be interpreted as indicators of inappropriate care per se, but rather as signals of contextual or data-related constraints, likely reflecting organizational factors, evolving clinical recommendations, and limitations inherent in available data sources. In particular, age-stratified analyses further indicate that part of the observed variation reflects clinically appropriate treatment selection rather than inappropriate underuse of care.

In general, the results of the pragmatic rapid reviews should be interpreted as operational planning anchors to support healthcare planning and care pathway implementation, rather than as definitive estimates. Although derived from a limited and partially heterogeneous body of literature, these estimates may still provide useful indications for policymakers and healthcare providers in the organization of prostate cancer care at the regional level. With regard to the proportion of patients undergoing magnetic resonance imaging for suspected prostate cancer who subsequently received a prostate biopsy, a substantial variability was observed across the included studies. Nevertheless, the median value was consistent with that reported in a large international multicenter study [10]. Concerning the proportion of biopsies resulting in a diagnosis of prostate cancer, most of the included studies showed largely consistent findings. However, a marked difference was observed between the detection rate referring to all malignant tumors—which was considered for the estimates presented in the present study—and that limited to clinically significant prostate cancer. This distinction is particularly relevant, as it may have a substantial impact on the estimation of healthcare needs. In the pragmatic rapid review on the distribution of newly diagnosed prostate cancer cases according to D’Amico risk categories, a high degree of heterogeneity was identified among the included studies, mainly due to differences in cohort selection and in the type of therapy delivered. Despite this heterogeneity, the median values and interquartile ranges were consistent with those reported in studies adopting alternative risk stratification systems [11,12,13,14,15]. Finally, the limited number of studies included in the pragmatic rapid review on the proportion of newly diagnosed patients who underwent radiotherapy, despite the consistency of their findings, may reflect a potential selection bias related to the inclusion criteria, search strategy, or the methodological constraints inherent to pragmatic rapid review approaches. In addition, these results do not enable a more detailed estimation according to treatment strategies tailored to disease stage and patient characteristics. Indeed, radiotherapy use in prostate cancer may vary depending on several factors, such as disease extent, recurrence, multimodal treatment strategies, and patient preferences, leading to variation in our operational planning anchors. The absence of formal risk of bias assessment and quantitative synthesis implies that the expected estimates should be interpreted as approximate and context-sensitive parameters rather than precise epidemiological measures.

The use of medians reduces the influence of extreme values but does not account for differences in study quality or sample size, which may affect the robustness of the estimates. In fact, a substantial degree of heterogeneity was observed across the studies included in the rapid reviews, reflecting differences in study populations, clinical settings, and diagnostic or therapeutic strategies. This variability directly affects the estimation of expected parameters and reinforces the need to interpret these values as approximate ranges rather than precise benchmarks [8]. In addition, selected indicators were calculated using different temporal windows in order to explore the timeliness of healthcare delivery along the prostate cancer care pathway. Timeliness represents a key quality dimension of cancer care and provides complementary information on service functioning beyond overall utilization rates. In the present analysis, these indicators suggest that, in general, the time elapsed between key steps of the diagnostic–therapeutic process and the delivery of healthcare services was longer than that recommended by international clinical guidelines. According to such guidelines, the clinical staging phase should be completed within approximately 30–45 days from the initial urological consultation, while definitive treatments such as surgical intervention and radiotherapy are generally expected to be initiated within 60 days from diagnosis or clinical decision-making. The use of multiple observation windows in this study was therefore intended to capture not only whether services were delivered, but also the extent to which their timing aligned with guideline-based standards of care, providing additional insights into potential organizational delays and the overall performance of the regional prostate cancer care pathway [16,17,18].

Overall, the results underscore the value of a methodology that entails the integration of population-based cancer registry data, healthcare administrative flows, and evidence from the literature to support healthcare planning and to inform the implementation and continuous monitoring of comprehensive oncological clinical pathways.

### 4.1. Comparison Between Observed and Expected Utilization of Healthcare Services

The distinction between observed and expected utilization represents a key methodological feature of this study. While population-based registry and administrative data enable accurate measurement of healthcare service use within the regional system, they do not provide a direct estimate of clinical need, as utilization patterns are shaped by organizational capacity, access to care, and local practice variations. The use of external literature therefore allows the estimation of expected utilization based on epidemiological evidence and clinical recommendations, independently of local constraints. This comparison provides a more informative framework for healthcare planning than either approach alone.

The comparison between observed and expected utilization revealed several relevant discrepancies across the diagnostic, staging, and therapeutic phases of the prostate cancer care pathway. These discrepancies can largely be explained by temporal, organizational, and data-related factors.

With regard to diagnostic imaging, the proportion of patients undergoing prostate MRI within the Regional Health Service was consistent across the two incident cohorts but substantially lower than expected based on published evidence. This finding is consistent with the timing of guideline updates: during the study period (2019–2022), multiparametric prostate MRI was formally incorporated into the updated regional diagnostic pathway only after the study period (2025), despite its growing clinical relevance documented in the literature [19,20]. Indeed, several studies have demonstrated the increasing role of MRI in improving the detection of clinically significant prostate cancer and in guiding biopsy decisions, particularly in younger patients and in those with equivocal PSA findings [19,20]. One possible explanation to the observed lower utilization therefore likely reflects a lag between emerging clinical evidence and its formal adoption within regional organizational frameworks, a phenomenon commonly described in the implementation of diagnostic innovations in healthcare systems [16]. However, other possible explanations cannot be excluded and may have contributed to this finding, including incomplete capture of MRI utilization in administrative data and the use of the private sector. Future analyses based on more recent cohorts will help to clarify the relative contribution of the lag between evidence generation and its adoption, as well as other potential factors.

Differences observed in biopsy-related indicators between the two cohorts are plausibly attributable to differing degrees of data consolidation. In the 2022 cohort, incident cases were identified primarily through administrative healthcare databases, without systematic linkage to pathology records, which represent the main source for accurate incidence dating in population-based cancer registries. This approach likely resulted in delayed assignment of the incidence date, often coinciding with surgical intervention rather than histological diagnosis, leading to an apparent reduction in the proportion of biopsies captured within predefined time windows. Similar methodological challenges related to case ascertainment, incidence dating, and misclassification have been widely reported in population-based studies relying on administrative data sources for cancer surveillance [21,22].

For staging imaging aimed at metastatic assessment, the observed proportions were lower than those expected based on operational planning anchors derived from the Italian literature. Nevertheless, this expected proportion should be interpreted cautiously, as the available literature did not provide a direct estimation of the proportion of patients requiring metastatic staging imaging. Therefore, the benchmark relied on a pragmatic approximation intended to provide a reference for health service planning and policymakers, rather than a direct translation of guideline recommendations, which are rarely based on a fixed threshold approach. International guidelines indicate that systemic staging examinations are required in a substantial proportion of newly diagnosed prostate cancer patients, particularly those with intermediate- to high-risk disease [17]. The discrepancies observed between cohorts—especially when narrower temporal windows around diagnosis were considered—further support the hypothesis that imprecision in incidence dating and incomplete capture of early diagnostic procedures play a relevant role, particularly in less consolidated datasets.

Regarding therapeutic interventions, the apparent underestimation of non-surgically treated patients in the 2022 cohort suggests incomplete capture of patients managed conservatively or treated exclusively with radiotherapy or systemic therapies, who may be less consistently recorded in hospital discharge databases. This issue is particularly relevant in prostate cancer, where treatment strategies vary widely according to clinical stage, risk stratification, patient age, and comorbidities, as documented in national observational studies [23,24]. As a consequence, administrative data may preferentially capture surgically treated patients, distorting the apparent distribution of care pathways. In addition, an updated linkage with pathology data could be used as a sensitivity analysis to evaluate the completeness and effectiveness of the monitoring system based on current healthcare administrative databases in comprehensively identifying incident cases.

Radiotherapy-related indicators were generally consistent between cohorts and aligned with evidence from national and international studies, which identify radiotherapy as a primary or complementary treatment option in a substantial proportion of patients, particularly those not undergoing surgery or those with biochemical recurrence or metastatic disease [24,25]. The higher use of radiotherapy among non-operated patients observed in this study is therefore expected and consistent with current clinical guidelines.

Nevertheless, comparisons between the 2019 and 2022 cohorts must be interpreted with extreme caution due to the profound impact of the COVID-19 pandemic on the healthcare ecosystem. During 2020–2022, regional healthcare delivery underwent unprecedented structural reorganizations, which severely affected diagnostic and therapeutic activity, prolonged clinical timelines, and potentially compromised the completeness and reporting speed of routine administrative data streams. Consequently, the observed variations between the pre-pandemic baseline (2019) and the 2022 cohort may partially reflect pandemic-induced systemic disruptions and data recording lags rather than true epidemiological shifts or long-term changes in clinical practice. While a formal analysis of the pandemic’s impact falls outside the scope of this study, this contextual confounding factor represents a critical caveat when comparing these two specific time periods [26].

The interpretation of discrepancies between observed and expected utilization requires careful consideration. Such differences should be interpreted as signals of system performance requiring contextual interpretation rather than direct proxies of underutilization. Rather, they may reflect a combination of factors. From this perspective, at least three non-mutually exclusive explanations should be considered when interpreting the observed–expected gap: (i) potential lower provision due to access or organizational constraints; (ii) clinically appropriate variation driven by age, comorbidity, stage, and patient preferences; (iii) methodological and data-related limitations affecting both observed and expected estimates. Disentangling these factors is essential to avoid misinterpretation of results and to support appropriate healthcare planning decisions.

The use of local data could be further extended to complement need-based estimates through stratified analyses, for example by age group, treatment patterns, or geographical area. Such analyses may help identify population subgroups or local contexts in which discrepancies between observed and expected utilization are particularly pronounced, thereby supporting more targeted planning interventions. In particular, age-stratified analyses suggest that variations in treatment patterns are strongly influenced by patient characteristics. Younger patients were more likely to undergo surgical treatment, whereas older patients more frequently received radiotherapy or more conservative management strategies. In the oldest age groups, lower treatment rates may reflect appropriate clinical decision-making, taking into account comorbidities, life expectancy, and patient preferences, rather than underutilization of care.

### 4.2. Comparison with Previous Studies and Implications for Healthcare Planning

Although several studies have described the incidence, prevalence, and treatment patterns of prostate cancer in Italy [7,27,28,29], to date, few studies have attempted to translate epidemiological data into explicit estimates of healthcare service demand across the care pathway [30,31,32]. In this respect, the present study proposes a structured, population-based and guideline-informed methodological framework for demand estimation, integrating different information sources previously used for oncology service planning and radiotherapy resource allocation, contributing to quantify the gap between expected and observed service utilization and to underscore strengths and limitations of the different sources of data [30,31,32].

The estimated demand for diagnostic imaging and radiotherapy exceeded observed service utilization, particularly for radiotherapy. Similar gaps between optimal and actual radiotherapy utilization have been reported in several European countries and are commonly interpreted as indicators of potential under-provision, organizational constraints, or barriers to access [33,34,35].

From a healthcare governance perspective, these findings highlight the value of demand estimation models as decision-support tools to identify divergences between clinical needs and available resources and to inform evidence-based planning and prioritization processes, as previously emphasized in health services research [33,34,35,36].

### 4.3. Strengths and Limitations

The main strength of this study lies in the development and application of a structured, transparent, and reproducible methodology for estimating healthcare service utilization in oncology, grounded in real-world population data and aligned with clinical guidelines and scientific literature. While prostate cancer in the Lazio region represents the application context of this study, the methodological approach was explicitly conceived to be transferable and adaptable to other oncological pathways and healthcare settings, in line with established models for cancer service planning and resource estimation [33,34,35].

From a broader perspective, this study underscores the importance of embedding demand estimation exercises within the continuous monitoring and revision of clinical pathways. As clinical recommendations evolve and healthcare systems adapt, dynamic and data-informed models are required to ensure alignment between clinical needs, organizational capacity, and policy objectives. In this context, the use of the Lazio Cancer Registry ensured accurate case definition and incidence dating for the consolidated cohort. Its integration with administrative healthcare flows and evidence from the literature represents a pragmatic and robust foundation for informed decision-making.

However, several limitations should be acknowledged. The rapid evolution of clinical recommendations—particularly with regard to radiotherapy indications and advanced imaging techniques—poses challenges to the use of published literature as a stable reference for expected demand. For radiotherapy this could become more relevant in more recent cohorts, due for example to its greater use in localized disease and postoperative salvage strategies. Furthermore, it has to be noted that the increasing use of modern radiotherapy approaches, such as hypofractionation, SBRT, as well as PSMA-guided treatments, could impact on radiotherapy service capacity and patient volume. Monitoring the literature and having a continuous interaction with clinical experts is therefore essential to validate and periodically update the assumptions underlying the estimates.

Reliance on historical data limits the ability to fully capture recent organizational changes, such as the formal introduction of prostate MRI into the updated regional clinical pathway. As healthcare systems evolve rapidly, historical utilization patterns may only partially reflect current and future needs. Nonetheless, population-based cancer registries remain a cornerstone of healthcare planning, offering comprehensive and reliable data that can be systematically updated over time.

The potential lack of information on services delivered in the private healthcare sector (i.e., outside the regional public healthcare system) may lead to an underestimation of service volumes, particularly for diagnostic imaging. However, its overall impact on estimated service utilization is expected to be modest particularly because of the highly specialized and technology-intensive nature of advanced oncological imaging and radiotherapy services, which are predominantly delivered within accredited public or network-affiliated centers, due to the substantial costs associated with these procedures [31,35]. Therefore, although we cannot exclude the missing information due to services provided in the private sector, we hypothesized that our data sources (HDRs and OSCIS) capture the vast majority of radiotherapy procedures delivered to the regional population.

The pragmatic rapid review component was not designed as a full systematic review. The lack of formal risk of bias assessment, absence of study weighting, and use of simplified synthesis methods (e.g., medians rather than meta-analysis) may have influenced the estimated parameters. In particular, studies with different methodological quality contributed equally to the summary estimates, and potential biases in the original studies were not formally accounted for. As a result, the expected utilization values should be interpreted as pragmatic approximations intended to support planning rather than definitive benchmarks. In addition, it should be noted that the radiotherapy estimates were based on only three included studies. Although a large number of articles were screened, only three met all the inclusion criteria, which represents a relevant limitation of this specific estimation. These limitations may partially explain discrepancies between expected and observed utilization, as the expected values are subject to uncertainty related to the underlying evidence base. In fact, the expected estimates are subject to uncertainty due to the heterogeneity of the underlying literature. Although median values were used to provide robust central estimates, the wide range observed across studies indicates that these parameters may vary substantially depending on the clinical and organizational context. While sensitivity analyses confirmed the overall direction of the results, the magnitude of the gap between expected and observed utilization should be interpreted with caution. Nevertheless, this approach is consistent with the purpose of rapid reviews in health policy contexts, where timely and context-specific evidence is required to inform decision-making [8]. Additionally, the expected-demand benchmarks are based on clinical risk distributions (D’Amico classification) sourced from the literature rather than directly measured in the entire study cohort.

Although literature-based estimates provide an approximation of expected need, they may not fully capture local epidemiological and organizational characteristics. In fact, a key limitation of the current study is that the proposed framework has not yet undergone formal local calibration or validation against clinically adjudicated treatment appropriateness. While literature-based estimates provide a valuable operational starting point, they inherently lack the granularity to account for specific local epidemiological patterns, such as the actual distribution of comorbidities, or regional organizational constraints that directly influence clinical decision-making. Consequently, these literature-derived approximations may not perfectly reflect the true clinical appropriateness within the Lazio Region. Future methodological developments will require a formal calibration phase, involving direct validation against clinical medical records and consensus-building with local multidisciplinary tumor boards (e.g., urologists, radiation oncologists, and oncologists) to refine the parameters and ensure they align with real-world, context-specific appropriateness of care.

Limitations in procedural coding within administrative databases hinder the precise identification of site-specific imaging and radiotherapy indications, a well-recognized issue in health services research that warrants further standardization efforts. Although the coding algorithms employed are based on established regional monitoring standards, they have not undergone formal validation against clinical medical records for the specific cohorts under study. This could lead to a potential underestimation or overestimation of certain procedures, particularly for those codes that are not uniquely specific to prostate cancer care. However, the consistency of our findings with regional clinical trends suggests that the impact of such misclassification is likely limited.

Finally, this analysis did not explicitly account for patients with disease recurrence or for the chronic clinical course that prostate cancer may assume in a subset of patients, potentially leading to sustained demand for diagnostic imaging and radiotherapy services over time [37].

## 5. Conclusions

This study presents a population-based and literature-informed framework for comparing observed and expected utilization of diagnostic imaging and radiotherapy in prostate cancer care. The application in the Lazio Region highlights differences between literature-derived benchmark estimates and real-world service use, underscoring the value of integrated approaches combining registry data, administrative healthcare information, and evidence from the literature to support healthcare planning. While developed in a specific regional context, the framework may be adapted to other settings, provided that local validation and contextual recalibration are performed.

## Figures and Tables

**Table 1:** Age distribution of patients in the 2019 and 2022 cohorts.

Cohort	2019 (N = 3047)	2022 (N = 3254)
	n (%)	
20–54	132 (4.3)	126 (3.9)
55–64	649 (21.3)	748 (23.0)
65–74	1239 (40.7)	1477 (45.4)
75–84	839 (27.5)	768 (23.6)
85+	188 (6.2)	135 (4.1)
Median (IQR)	71.15 (12.5)	70.36 (11.2)

## Data Availability

The aggregated data supporting the findings of this study are reported within the manuscript and its Supplementary Materials. Individual-level administrative healthcare and cancer registry data used for the analyses cannot be publicly shared because of legal and privacy restrictions under Italian and European data protection regulations. Access to aggregated data and additional methodological information may be provided upon reasonable request to the Department of Epidemiology of the Lazio Regional Health Service, for researchers who meet the criteria for access to confidential health data under applicable regulations. Requests may be directed to: Paola Michelozzi, Head of the Department of Epidemiology of the Lazio Regional Health Service, Rome, Italy (email: dipepi@deplazio.it).

## Supplementary Materials

The following supporting information can be downloaded at: https://www.mdpi.com/article/10.3390/healthcare14111597/s1, Table S1: the research strings; Table S2: outcomes described and defined according to the ICD-9-CM procedure codes (primary or secondary) for the HDR and the Italian nomenclature of outpatient specialist assistance for the OSCIS; Table S3: the observation windows related to each outcome; Figure S1: PRISMA 2020 flow diagram of rapid review n. (1) the proportion of patients with MRI performed for suspected prostate cancer who subsequently underwent prostate biopsy; Figure S2: PRISMA 2020 flow diagram of rapid review n. (2) the proportion of biopsies resulting in a diagnosis of prostate cancer; Figure S3: PRISMA 2020 flow diagram of rapid review n. (3) the distribution of newly diagnosed prostate cancer cases according to D’Amico risk categories (low, intermediate, and high); Figure S4: PRISMA 2020 flow diagram of rapid review n. (4) the proportion of newly diagnosed patients who underwent radiotherapy; Table S4: extracted estimates of rapid review n. (1) the proportion of patients with MRI performed for suspected prostate cancer who subsequently underwent prostate biopsy; Table S5: extracted estimates of rapid review n. (2) the proportion of biopsies resulting in a diagnosis of prostate cancer; Table S6: extracted estimates of rapid review n. (3) the distribution of newly diagnosed prostate cancer cases according to D’Amico risk categories (low, intermediate, and high); Table S7: extracted estimates of rapid review n. (4) the proportion of newly diagnosed patients who underwent radiotherapy; Table S8: Median, Interquartile range (IQR) and minimum and maximum values of the included articles in PIRADS score, biopsy and D’Amico score reviews; Table S9: diagnostic and treatment pathways of the 2019 and 2022 cohorts, stratified by age class. Median values are expressed in days; Figure S5: surgical treatment within 365 days post-diagnosis, stratified by age class in the two cohorts; Figure S6: radiotherapy within 365 days post-diagnosis, stratified by age class in the two cohorts.

## Abbreviations

The following abbreviations are used in this manuscript:

MRI	Magnetic Resonance Imaging
RT	Radiotherapy
IQR	Interquartile Range
LRCR	Lazio Region Cancer Registry
HIS	Healthcare Information System
HDR	Hospital Discharge Records
OSCIS	Outpatient Specialist Care Information System
95% CI	Ninety-five percent confidence intervals
