# Supplementary material for: Observed Versus Expected Use of Diagnostic Imaging and Radiotherapy in Prostate Cancer in the Lazio Region (Italy): An Integrated Population-Based and Literature-Informed Framework for Healthcare Planning
Source: Healthcare (Basel). 2026 Jun 5;14(11):1597. doi: 10.3390/healthcare14111597 (PMC13256337; doi:10.3390/healthcare14111597)

SUPPLEMENTARY MATERIALS

Table S1. The research strings.

**Objective**	**String**
The proportion of patients with MRI performed for suspected prostate cancer who subsequently underwent prostate biopsy	(("PI-RADS"[Title/Abstract] OR PIRADS[Title/Abstract] OR "Prostate Imaging Reporting and Data System"[Title/Abstract] OR "Prostate Imaging Reporting and Data System"[MeSH] OR (("Magnetic Resonance Imaging"[MeSH] OR MRI[Title/Abstract] OR mpMRI[Title/Abstract]) AND ("Prostate Neoplasms"[MeSH] OR prostate cancer[Title/Abstract]))) AND (distribution[Title/Abstract] OR prevalence[Title/Abstract] OR proportion[Title/Abstract] OR frequency[Title/Abstract] OR rate*[Title/Abstract] OR epidemiolog*[Title/Abstract] OR percentage[Title/Abstract] OR "Prevalence"[MeSH] OR "Epidemiology"[Subheading]) AND (Italy[Affiliation] OR Italy[Title/Abstract] OR Italian[Title/Abstract] OR Italia[Title/Abstract] OR "Italy"[MeSH])) Filters: from 2016/1/1 - 2026/1/31
The proportion of biopsies resulting in a diagnosis of prostate cancer	(("Prostatic Neoplasms"[Mesh] OR prostate cancer[Title/Abstract]) AND ("Biopsy"[Mesh] OR prostate biopsy[Title/Abstract] OR prostate biopsies[Title/Abstract]) AND (prevalence[Title/Abstract] OR proportion[Title/Abstract] OR percentage[Title/Abstract] OR positive rate[Title/Abstract] OR detection rate[Title/Abstract]) AND (Italy[Affiliation] OR Italy[Title/Abstract] OR Italian[Title/Abstract])) Filters: from 2016/1/1 - 2026/1/31
The distribution of newly diagnosed prostate cancer cases according to D'Amico risk categories (low, intermediate, and high)	((("Prostatic Neoplasms"[MeSH] OR "prostate cancer"[Title/Abstract] OR "prostatic neoplasms"[Title/Abstract]) AND ("Risk Stratification"[MeSH] OR "risk group*"[Title/Abstract] OR "risk classification"[Title/Abstract] OR "intermediate risk"[Title/Abstract] OR "high risk"[Title/Abstract] OR "intermediate-risk"[Title/Abstract] OR "high-risk"[Title/Abstract] OR "D'Amico"[Title/Abstract] OR "EAU risk"[Title/Abstract] OR "NCCN risk"[Title/Abstract]) AND (prevalence[Title/Abstract] OR proportion*[Title/Abstract] OR percent*[Title/Abstract] OR distribution[Title/Abstract])) AND (Italy[Affiliation] OR Italy[Title/Abstract] OR Italian[Title/Abstract])) Filters: from 2016/1/1 - 2026/1/31

The proportion of newly diagnosed patients who underwent radiotherapy	((("Prostatic Neoplasms"[MeSH] OR "prostate cancer"[Title/Abstract] OR "prostatic neoplasms"[Title/Abstract]) AND ("Radiotherapy"[MeSH] OR "radiation therapy"[Title/Abstract] OR radiotherapy[Title/Abstract] OR irradiation[Title/Abstract]) AND ("Health Services Utilization"[MeSH] OR utilization[Title/Abstract] OR utilisation[Title/Abstract] OR proportion*[Title/Abstract] OR percent*[Title/Abstract] OR prevalence[Title/Abstract] OR rate*[Title/Abstract] OR uptake[Title/Abstract] OR pattern*[Title/Abstract])) AND (Italy[Affiliation] OR Italy[Title/Abstract] OR Italian[Title/Abstract]))

Table S2. Outcomes described and defined according to the ICD-9-CM procedure codes (primary or secondary) for the HDR and the Italian nomenclature of outpatient specialist assistance for the OSCIS database.

Outcomes	Source	Description	Codes
**Surgical Intervention**	HDR	Radical prostate resection / prostatectomy	60.3, 60.4, 60.5, 60.61, 60.62, 60.69
	HDR	Transurethral resection of the prostate (TURP)	60.21, 60.29, 60.96, 60.97
**Magnetic Resonance Imaging**	OSCIS	MRI of the lower abdomen and pelvic cavity	88.95.4
	OSCIS	MRI of the lower abdomen and pelvic cavity with and without contrast	88.95.5
	HDR	MRI of pelvis, prostate, and bladder	88.95
**Prostate Biopsy**	OSCIS	Transperineal or transrectal prostate biopsy	60.11
	OSCIS	Ultrasound-guided prostate biopsy	60.11.1
	HDR	Prostate biopsy (transrectal, transperineal, or transurethral approach)	60.11
	HDR	Open prostate biopsy	60.12
**Imaging for Metastatic Staging**	HDR	Bone scintigraphy	92.14
	HDR	Whole-body scintigraphy	92.18
	HDR	Other computed tomography-Excludes CT of abdomen (88.01), head (87.03), kidney (87.71), chest (87.41)	88.38
	HDR	Computed tomography of the abdomen	88.01
	HDR	Computed tomography of the head	87.03
	HDR	Computed tomography of the kidney-Excludes eye (95.13)	87.71
	HDR	Computed tomography of the chest	87.41
	OSCIS	Whole-body bone marrow scintigraphy	92.05.6
	OSCIS	Segmental or joint bone scintigraphy	92.14.1
	OSCIS	Segmental or joint multiphasic bone scintigraphy	92.14.2
	OSCIS	Whole-body scintigraphy with positive indicators	92.18.1
	OSCIS	Bone or joint scintigraphy	92.18.2
	OSCIS	Whole-body scintigraphy with immunologic or receptor tracers	92.18.5
	OSCIS	Whole-body PET (tomoscintigraphy)	92.18.6
	OSCIS	Multiphasic whole-body bone scintigraphy	92.18.7
	OSCIS	Whole-body CT	88.38.9
**Radiotherapy**	OSCIS	Hyperthermia for tumor treatment	99.85

OSCIS	Injection of contrast agent for radiotherapy simulation CT	38.99.1
OSCIS	Injection of contrast agent for radiotherapy simulation MRI	38.99.2
OSCIS	Roentgenterapy	92.21.1
OSCIS	Telecobalt therapy with fixed field or two opposing fields	92.23.1
OSCIS	Telecobalt therapy with multiple moving fields	92.23.2
OSCIS	Telecobalt therapy with flash technique	92.23.3
OSCIS	Teletherapy with linear accelerator, fixed field or two opposing fields	92.24.1
OSCIS	Teletherapy with linear accelerator, multiple moving fields	92.24.2
OSCIS	Teletherapy with linear accelerator, flash technique	92.24.3
OSCIS	Stereotactic radiotherapy	92.24.4
OSCIS	Stereotactic radiotherapy, subsequent sessions	92.24.5
OSCIS	Conformal radiotherapy	92.24.6
OSCIS	Intensity-modulated radiotherapy (IMRT)	92.24.7
OSCIS	Teletherapy with electrons, one or more fixed fields	92.25.1
OSCIS	Total skin electron irradiation (TSEI/TSEBI)	92.25.2
OSCIS	Endocavitary brachytherapy with remote loading (HDR)	92.27.1
OSCIS	Surface brachytherapy (HDR), per session and treated lesion	92.27.3
OSCIS	Interstitial brachytherapy with remote loading (HDR)	92.27.4
OSCIS	Contact betatherapy	92.27.5
OSCIS	Endocavitary therapy	92.28.3
OSCIS	Monoclonal antibody therapy (Up to 185 MBq)	92.28.4
OSCIS	Monoclonal antibody therapy (For each additional 185 MBq)	92.28.5
OSCIS	Palliative therapy for bone metastasis pain	92.28.6
OSCIS	Target volume identification and simulation	92.29.1
OSCIS	Target volume identification and simulation	92.29.2
OSCIS	Target volume identification and simulation	92.29.3
OSCIS	Physical-dosimetric study	92.29.4
OSCIS	Physical-dosimetric study with computer on CT scans	92.29.5
OSCIS	In vivo dosimetry	92.29.6
OSCIS	Personalized shielding	92.29.7
OSCIS	Personalized immobilization system	92.29.8
OSCIS	Preparation of shaped compensators	92.29.9
OSCIS	Interstitial brachytherapy with permanent implant	92.27.2

OSCIS	Teletherapy with intensity modulation (IMRT) – multiple arcs or helical, IGRT; short cycle ≤5 sessions	92.47.8
OSCIS	Teletherapy with intensity modulation (IMRT) – multiple arcs or helical, IGRT; long cycle >5 sessions	92.4
HDR	Therapeutic radiology and nuclear medicine	92.2

Table S3. The observation windows related to each outcome.

Outcomes	Observation Windows
**Magnetic Resonance Imaging**	Within 60 days before the index date
	Within 180 days before the index date
**Biopsy**	Within 60 days before the index date
	Within 180 days before the index date
	On the index date
	Within 180 days after the index date
**Imaging for Metastatic Staging**	Within 60 days before and 180 days after the index date
**Surgical Intervention**	Within 60 days after the index date
	Within 90 days after the index date
	Within 365 days after the index date
**Radiotherapy (with or without surgery)**	Within 365 days after the index date
**Adjuvant Radiotherapy**	Within 180 days after the date of surgical intervention
	Within 365 days after the date of surgical intervention
**Radiotherapy without Surgery**	Within 60 days after the index date
	Within 180 days after the index date
	Within 365 days after the index date

Figure S1. PRISMA 2020 flow diagram of rapid review n. 1) the proportion of patients with MRI performed for suspected prostate cancer who subsequently underwent prostate biopsy.

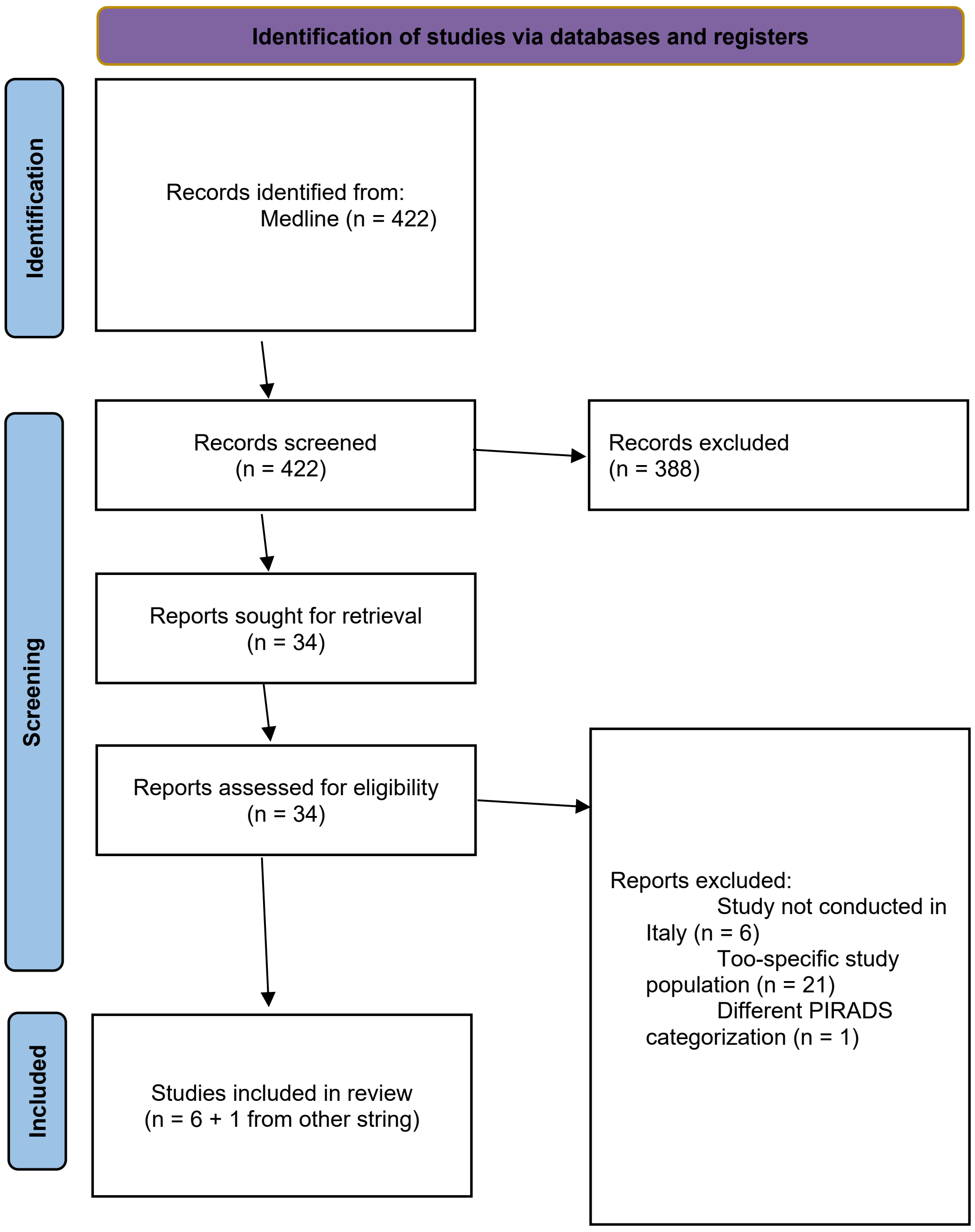

From: Page MJ, McKenzie JE, Bossuyt PM, Boutron I, Hoffmann TC, Mulrow CD, et al. The PRISMA 2020 statement: an updated guideline for reporting systematic reviews. BMJ 2021;372:n71. doi: 10.1136/bmj.n71

For more information, visit: http://www.prisma-statement.org/

Figure S2. PRISMA 2020 flow diagram of rapid review n. 2) the proportion of biopsies resulting in a diagnosis of prostate cancer.

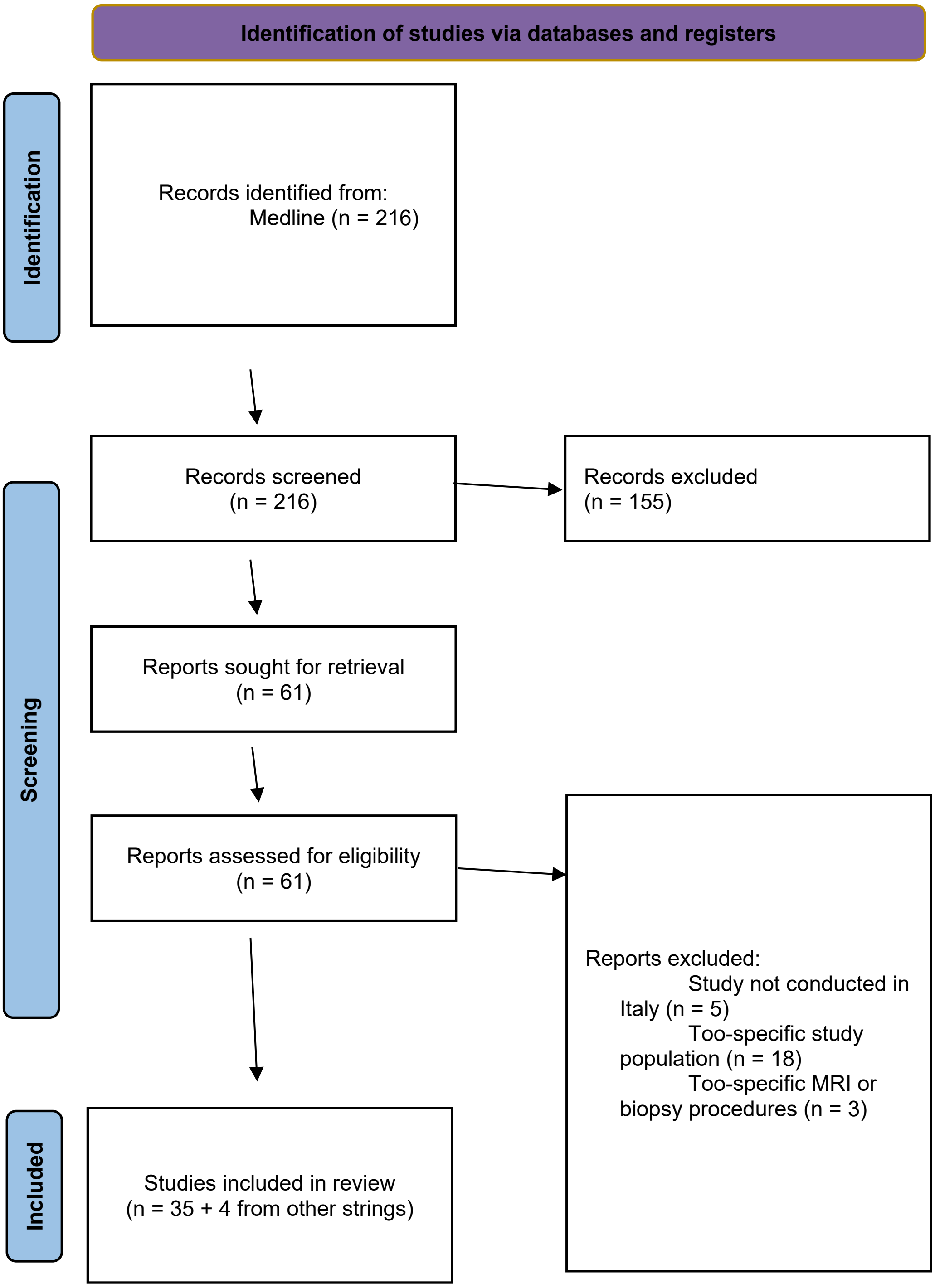

From: Page MJ, McKenzie JE, Bossuyt PM, Boutron I, Hoffmann TC, Mulrow CD, et al. The PRISMA 2020 statement: an updated guideline for reporting systematic reviews. BMJ 2021;372:n71. doi: 10.1136/bmj.n71

For more information, visit: http://www.prisma-statement.org/

Figure S3. PRISMA 2020 flow diagram of rapid review n. 3) the distribution of newly diagnosed prostate cancer cases according to D'Amico risk categories (low, intermediate, and high).

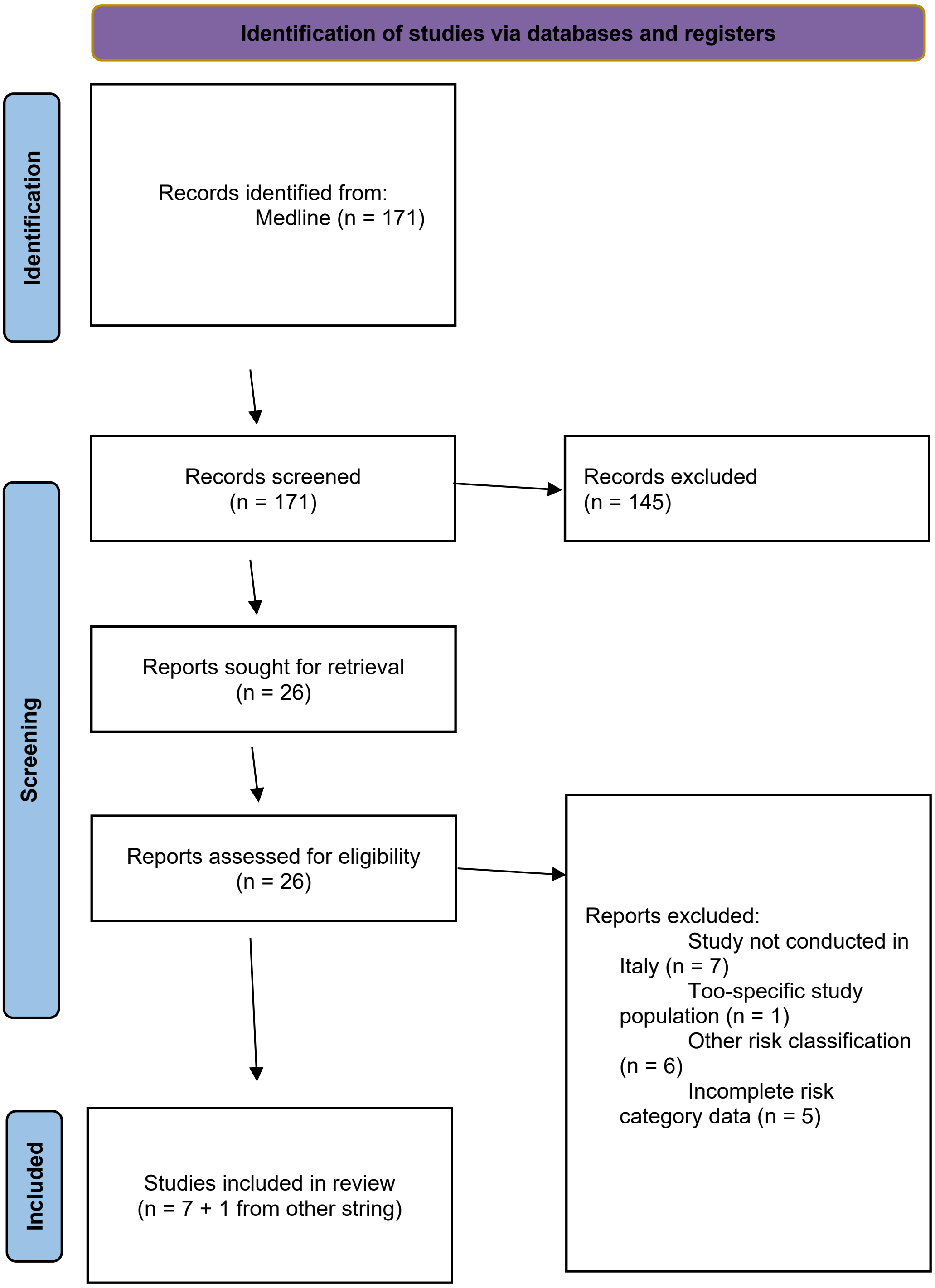

From: Page MJ, McKenzie JE, Bossuyt PM, Boutron I, Hoffmann TC, Mulrow CD, et al. The PRISMA 2020 statement: an updated guideline for reporting systematic reviews. BMJ 2021;372:n71. doi: 10.1136/bmj.n71

For more information, visit: http://www.prisma-statement.org/

Figure S4. PRISMA 2020 flow diagram of rapid review n. 4) the proportion of newly diagnosed patients who underwent radiotherapy.

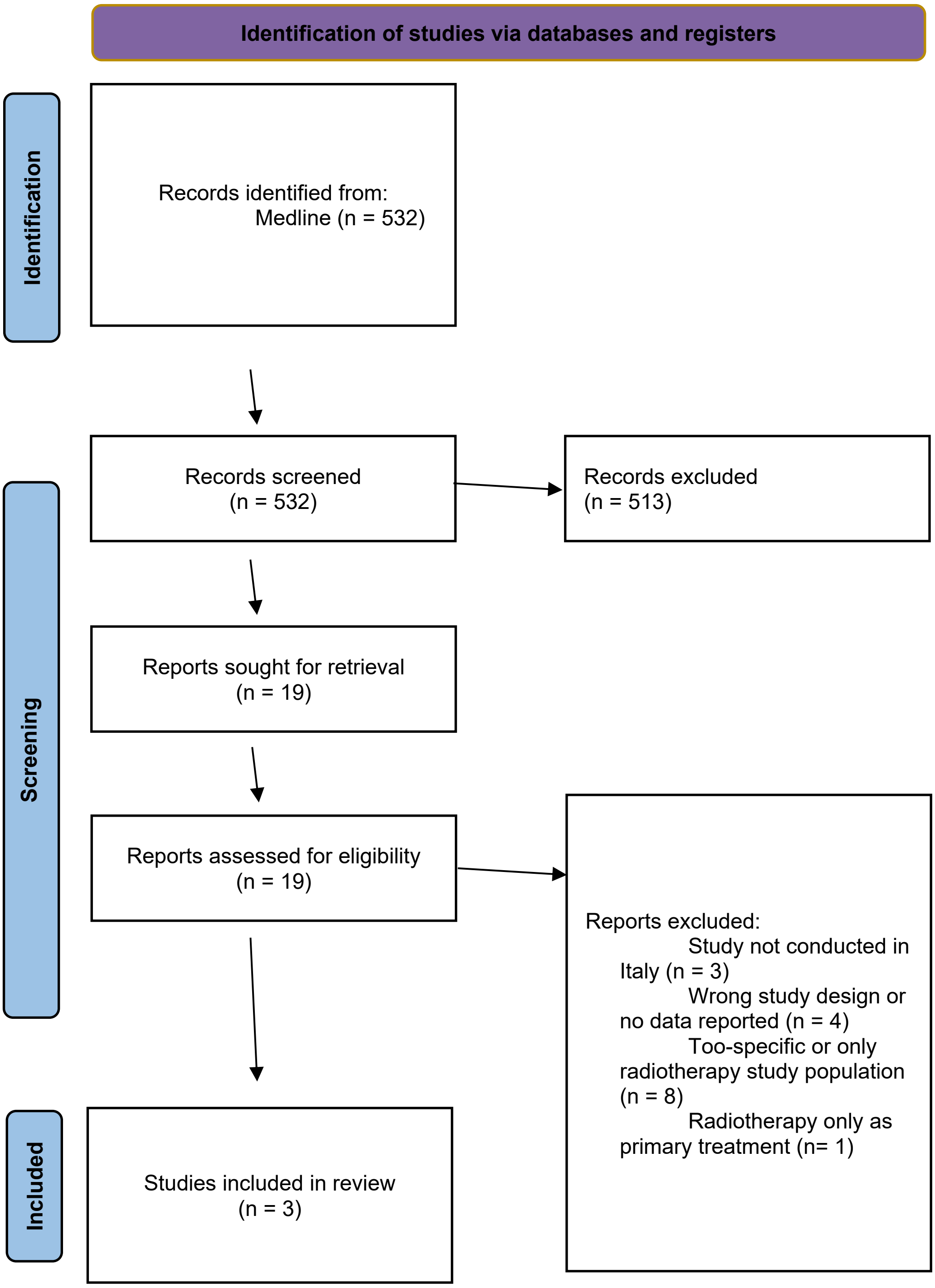

From: Page MJ, McKenzie JE, Bossuyt PM, Boutron I, Hoffmann TC, Mulrow CD, et al. The PRISMA 2020 statement: an updated guideline for reporting systematic reviews. BMJ 2021;372:n71. doi: 10.1136/bmj.n71

For more information, visit: http://www.prisma-statement.org/

Table S4. Extracted estimates of rapid review n. 1) the proportion of patients with MRI performed for suspected prostate cancer who subsequently underwent prostate biopsy.

Author	Publication year	% PIRADS < 3	% PIRADS ≥ 3
Girometti R et al	2022	29.0	71.0
Brembilla G et al	2020	45.0	55.0
Massanova M et al	2024	10.5	89.5
Panebianco V et al	2018	31.0	69.0
Luzzago S et al	2021	55.0	45.0
Falagario UG et al	2021	15.6	84.4
From other string			
Luzzago S et al*	2019	15.0	74.0
Luzzago S et al*	2019	51.5	37.5

*Two estimates from the same paper. In Luzzago et al. (2019), a subset of examinations (11%) reported an index lesion without a PI-RADS category assignment. For this reason, the percentages of PI-RADS <3 and PI-RADS ≥3 are calculated on the full study denominator but are not complementary and do not sum to 100%.

Table S5. Extracted estimates of rapid review n. 2) the proportion of biopsies resulting in a diagnosis of prostate cancer.

Author	Publication year	% PCA	% csPCA
Cannoletta D et al	2026	69.6	49.9
Sattin C et al	2025	62.3	35.8
Tulone G et al	2025	65.0	49.4
Amparore D et al	2025	60.3	56.2
Oderda M et al	2025	64.0	58.8
Carletti F et al	2025	62.3	45.5
Russo T et al	2025	68.0	47.0
Girometti R et al	2025		30.3
Massanova M et al	2024	67.7	35.7
Turchi B et al	2024	49.3	37.0
Girometti R et al	2024	50.0	38.0
Pellegrino F et al	2024		50.0
Barone B et al	2023	52.2	36.7
Oderda M et al	2023	63.2	58.7
Brembilla G et al	2023	61.0	43.0
Novara G et al	2023	64.0	49.0
Droghetti M et al	2023	55.5	43.7
Ninivaggi A et al	2023	52.7	30.3
Girometti R et al	2022		43.9
Del Monte M et al	2022	62.8	43.5
Fulco A et al	2021	43.0	27.2
Borghesi M et al	2021	73.4	62.7
Bertolo R et al	2021	52.3	44.5
Ferriero M et al	2021	59.6	30.9
Lughezzani G et al	2021	48.7	36.3
Luzzago S et al	2021	70.6	35.3
D'agostino D et al	2021	60.4	41.6
Benelli A et al	2020	54.1	34.8
Cecchini S et al	2020	54.4	34.8
Brembilla G et al	2020	78.8	32.6
Cattarino S et al	2019	53.0	26.0
Mantica G et al	2019	59.6	
Stabile A et al	2018	64.8	53.3
Stabile A et al	2018	65.3	53.3
Oderda M et al	2016	52.0	24.0
From other strings			
Stabile A et al	2023	54.0	40.0
Tafuri A et al	2021	45.4	35.4
Brancato V et al	2020	64.9	34.2
Schiavina R et al	2017	45.7	34.3

Table S6. Extracted estimates of rapid review n. 3) the distribution of newly diagnosed prostate cancer cases according to D'Amico risk categories (low, intermediate, and high).

Author	Publication year	Score	% Low risk	% Intermediate risk	% High risk
Cozzi S et al	2023	D'Amico classification	21.0	46.4	32.6
Tafuri A et al	2022	D'Amico classification	48.0	38.0	14.0
Sortino G et al	2021	D'Amico classification	22.4	52.8	24.8
Tafuri A et al	2020	D'Amico classification	24.0	52.6	23.4
Afferi L et al	2021	D'Amico classification	22.7	62.3	15.0
Porcaro AB et al*	2017 and 2018	D'Amico classification	38.8	-	-
From other string					
Antonelli et al	2020	D'Amico classification	27.6	44.7	26.6
Other risk classification					
van den Bergh R et al**	2017	For the current study, patients were stratified as suitable or unsuitable for AS and therefore the risk groups presented above differ slightly from the more commonly used d'Amico criteria	18,0	66,0	15,0
Porcaro AB et al	2021	EAU risk class		53,4	
Oderda M et al***	2017	EAU risk class	25,0	46,1	28,8
Vicentini M et al<br>Trama A et al****	2017<br>2016	National Comprehensive Cancer Network (adapted)	34,0	26,0	26,0
Di Muzio NG et al	2016	National Comprehensive Cancer Network (adapted)	37,0	25,0	28,0

*The study reported only the proportion of low-risk cases.
** Slightly different from the D'amico criteria
*** Clinical percentages were reported. Pathological percentages were 4.0%, 48.0% and 48.0% for low, intermediate and high risk, respectively.
**** Metastatic percentage at diagnosis were also reported (7.0%)

Table S7. Extracted estimates of rapid review n. 4) the proportion of newly diagnosed patients who underwent radiotherapy.

Author	Publication year	% Radiotherapy
Bugliome M et al	2019	42.4
Antonelli et al	2020	39.4
Urso L et al	2022	33.3

Table S8. Median, Interquartile range (IQR) and minimum and maximum values of the included articles in PIRADS score, biopsy and D’amico score reviews.

	PIRADS score	Positive biopsy		D’amico score		
	% PIRADS ≥ 3	% PC*	% csPC**	% Low risk	% Intermediate risk	% High risk
Median	70.0	60.4	39.0	24.0	49.5	24.1
25°	52.5	52.6	34.8	22.6	45.1	17.1
75°	76.6	64.8	48.5	33.2	52.8	26.2
Min	37.5	43.0	24.0	21.0	38.0	14.0
Max	89.5	78.8	62.7	48.0	62.3	32.6

*Prostate cancer

** Clinically significant prostate cancer

Table S9. Diagnostic and treatment pathways of the 2019 and 2022 cohorts, stratified by age class. Median values are expressed in days.

2019 cohort	20-85+					20-54		55-64		65-74		75-84		85+	
	n	N	Median	(%)	95% CI	(%)	95% CI	(%)	95% CI	(%)	95% CI	(%)	95% CI	(%)	95% CI
MRI 60d pre-Dx	271	3,047	32	(8.89)	[7.93 - 9.96]	(10.61)	[6.42 - 17.02]	(9.71)	[7.66 - 12.23]	(9.69)	[8.16 - 11.46]	(8.22)	[6.55 - 10.28]	(2.66)	[1.14 - 6.07]
MRI 180d pre-Dx	563	3,047	62	(18.48)	[17.14 - 19.89]	(18.18)	[12.53 - 25.63]	(20.49)	[17.57 - 23.77]	(21.07)	[18.89 - 23.42]	(16.45)	[14.09 - 19.11]	(3.72)	[1.82 - 7.48]
BIOPSY 0d pre-Dx	1,200	3,047	0	(39.38)	[37.66 - 41.13]	(36.36)	[28.65 - 44.85]	(38.37)	[34.70 - 42.17]	(39.31)	[36.62 - 42.05]	(46.13)	[42.78 - 49.51]	(15.43)	[10.96 - 21.28]
BIOPSY 60d pre-Dx	1,901	3,047	0	(62.39)	[60.65 - 64.09]	(55.30)	[46.79 - 63.52]	(63.48)	[59.71 - 67.10]	(63.60)	[60.88 - 66.23]	(68.89)	[65.68 - 71.93]	(26.60)	[20.79 - 33.33]
BIOPSY 180d pre-Dx	1,989	3,047	0	(65.28)	[63.57 - 66.95]	(57.58)	[49.05 - 65.68]	(67.95)	[64.26 - 71.43]	(67.23)	[64.57 - 69.79]	(70.20)	[67.02 - 73.20]	(26.60)	[20.79 - 33.33]
BIOPSY 180d post-Dx	1,291	2,837	0	(45.51)	[43.68 - 47.34]	(39.39)	[31.47 - 47.92]	(41.72)	[37.96 - 45.58]	(43.89)	[41.12 - 46.70]	(53.31)	[49.78 - 56.80]	(35.37)	[25.89 - 46.16]
IMAGING 180d post-Dx	1,159	2,837	40	(40.85)	[39.06 - 42.67]	(29.55)	[22.43 - 37.82]	(32.81)	[29.29 - 36.54]	(39.36)	[36.64 - 42.14]	(50.97)	[47.45 - 54.49]	(48.78)	[38.26 - 59.41]
SURGERY 60d post-Dx	617	2,900	0	(21.28)	[19.82 - 22.80]	(29.55)	[22.43 - 37.82]	(33.85)	[30.31 - 37.58]	(22.61)	[20.36 - 25.04]	(9.01)	[7.22 - 11.20]	(10.31)	[5.70 - 17.95]
SURGERY 90d post-Dx	796	2,876	1	(27.68)	[26.07 - 29.34]	(42.42)	[34.32 - 50.95]	(43.41)	[39.64 - 47.26]	(30.39)	[27.88 - 33.03]	(9.94)	[8.03 - 12.23]	(11.11)	[6.15 - 19.26]
SURGERY 365d post-Dx	1,305	2,769	68	(47.13)	[45.27 - 48.99]	(61.54)	[52.96 - 69.46]	(69.72)	[66.03 - 73.17]	(54.87)	[52.03 - 57.67]	(16.24)	[13.75 - 19.07]	(12.16)	[6.53 - 21.53]
RT 365d post-Dx	637	2,769	198	(23.00)	[21.48 - 24.61]	(13.85)	[8.94 - 20.83]	(10.73)	[8.55 - 13.38]	(21.48)	[19.24 - 23.90]	(39.11)	[35.65 - 42.67]	(8.11)	[3.77 - 16.58]
Adjuvant RT 180d post-Op	109	1,313	134	(8.30)	[6.93 - 9.92]	(10.00)	[5.15 - 18.51]	(6.55)	[4.60 - 9.24]	(7.91)	[6.09 - 10.23]	(16.39)	[10.87 - 23.97]	(0.00)	[0.00 - 25.88]
Adjuvant RT 365d post-Op	238	1,305	190	(18.24)	[16.24 - 20.42]	(20.00)	[12.70 - 30.05]	(13.35)	[10.49 - 16.84]	(19.27)	[16.43 - 22.46]	(30.83)	[23.27 - 39.58]	(0.00)	[0.00 - 29.91]
RT without surgery 60d post-Dx	54	1,584	37.5	(3.41)	[2.62 - 4.42]	(3.85)	[1.06 - 12.98]	(3.45)	[1.68 - 6.95]	(3.70)	[2.44 - 5.60]	(2.96)	[1.92 - 4.53]	(4.65)	[1.82 - 11.36]
RT without surgery 180d post-Dx	341	1,524	115	(22.38)	[20.35 - 24.54]	(21.15)	[12.24 - 34.03]	(12.18)	[8.33 - 17.49]	(25.77)	[22.30 - 29.56]	(24.50)	[21.35 - 27.95]	(5.63)	[2.21 - 13.61]
RT without surgery 365d post-Dx	591	1,464	161	(40.37)	[37.88 - 42.90]	(28.00)	[17.47 - 41.67]	(29.69)	[23.67 - 36.50]	(42.75)	[38.63 - 46.97]	(45.88)	[41.99 - 49.82]	(9.23)	[4.30 - 18.71]

2022 cohort	20-85+					20-54		55-64		65-74		75-84		85+	
	n	N	Median	(%)	95% CI	(%)	95% CI	(%)	95% CI	(%)	95% CI	(%)	95% CI	(%)	95% CI
MRI 60d pre-Dx	217	3,254	38	(6.67)	[5.86 - 7.58]	(6.82)	[5.22 - 8.85]	(6.82)	[5.20 - 8.90]	(6.77)	[5.60 - 8.17]	(7.03)	[5.43 - 9.06]	(2.96)	[1.16 - 7.37]
MRI 180d pre-Dx	495	3,254	68	(15.21)	[14.02 - 16.49]	(16.58)	[14.08 - 19.41]	(16.58)	[14.10 - 19.40]	(15.91)	[14.13 - 17.86]	(14.45)	[12.14 - 17.12]	(5.93)	[3.03 - 11.26]
BIOPSY 0d pre-Dx	650	3,254	0	(19.98)	[18.64 - 21.38]	(16.98)	[14.46 - 19.84]	(16.98)	[14.50 - 19.80]	(20.11)	[18.14 - 22.23]	(25.52)	[22.56 - 28.72]	(8.15)	[4.61 - 14.00]
BIOPSY 60d pre-Dx	1,344	3,254	1	(41.30)	[39.62 - 43.00]	(39.57)	[36.13 - 43.12]	(39.57)	[36.10 - 43.10]	(40.76)	[38.28 - 43.29]	(46.88)	[43.37 - 50.41]	(26.67)	[19.93 - 34.70]
BIOPSY 180d pre-Dx	1,645	3,254	14	(50.55)	[48.84 - 52.27]	(51.20)	[47.62 - 54.77]	(51.2)	[47.60 - 54.80]	(50.17)	[47.62 - 52.72]	(54.17)	[50.63 - 57.66]	(29.63)	[22.58 - 37.81]
BIOPSY 180d post-Dx	696	3,120	0	(22.31)	[20.88 - 23.80]	(18.46)	[15.84 - 21.42]	(18.46)	[15.80 - 21.40]	(22.24)	[20.17 - 24.45]	(28.93)	[25.72 - 32.37]	(11.83)	[6.73 - 19.95]
IMAGING 180d post-Dx	1,087	3,120	37	(34.84)	[33.19 - 36.53]	(29.65)	[26.48 - 33.03]	(29.65)	[26.50 - 33.00]	(33.22)	[30.84 - 35.69]	(44.66)	[41.05 - 48.33]	(37.63)	[28.46 - 47.79]
SURGERY 60d post-Dx	1,200	3,181	0	(37.72)	[36.06 - 39.42]	(47.11)	[43.55 - 50.70]	(46.85)	[43.30 - 50.40]	(40.83)	[38.34 - 43.37]	(23.53)	[20.60 - 26.74]	(11.50)	[6.85 - 18.69]
SURGERY 90d post-Dx	1,380	3,156	0	(43.73)	[42.00 - 45.46]	(54.84)	[51.25 - 58.38]	(54.57)	[51.00 - 58.10]	(47.98)	[45.42 - 50.54]	(25.83)	[22.77 - 29.14]	(12.26)	[7.31 - 19.86]
SURGERY 365d post-Dx	1,741	3,051	0	(57.06)	[55.30 - 58.81]	(70.84)	[67.46 - 74.02]	(70.3)	[66.90 - 73.50]	(62.59)	[60.05 - 65.06]	(32.65)	[29.24 - 36.26]	(16.25)	[9.75 - 25.84]
RT 365d post-Dx	687	3,051	190	(22.52)	[21.07 - 24.03]	(15.40)	[12.96 - 18.19]	(15.26)	[12.80 - 18.00]	(18.81)	[16.87 - 20.92]	(40.41)	[36.79 - 44.13]	(15.00)	[8.79 - 24.41]
Adjuvant RT 180d post-Op	115	1,750	125	(6.57)	[5.50 - 7.83]	(5.18)	[3.59 - 7.43]	(5.22)	[3.60 - 7.50]	(6.79)	[5.32 - 8.63]	(7.56)	[4.77 - 11.77]	(0.00)	[0.00 - 20.39]
Adjuvant RT 365d post-Op	216	1,741	172	(12.41)	[10.94 - 14.04]	(11.73)	[9.24 - 14.78]	(11.63)	[9.10 - 14.70]	(11.62)	[9.68 - 13.89]	(15.70)	[11.51 - 21.05]	(7.69)	[1.37 - 33.31]
RT without surgery 60d post-Dx	54	1,427	44	(3.78)	[2.91 - 4.90]	(4.04)	[2.14 - 7.49]	(3.96)	[2.10 - 7.40]	(3.17)	[2.02 - 4.96]	(4.55)	[3.05 - 6.74]	(4.08)	[1.60 - 10.03]
RT without surgery 180d post-Dx	303	1,370	117	(22.12)	[20.00 - 24.39]	(16.74)	[12.40 - 22.22]	(16.44)	[12.20 - 21.80]	(17.45)	[14.51 - 20.85]	(32.85)	[28.83 - 37.15]	(11.54)	[6.19 - 20.50]
RT without surgery 365d post-Dx	467	1,310	160	(35.65)	[33.10 - 38.28]	(24.30)	[19.04 30.47]	(23.85)	[18.70 - 29.90]	(30.28)	[26.54 - 34.30]	(52.17)	[47.61 - 56.70]	(16.42)	[9.42 - 27.06]

209

Figure S5. Surgical treatment within 365 days post-diagnosis, stratified by age class in the two cohorts.

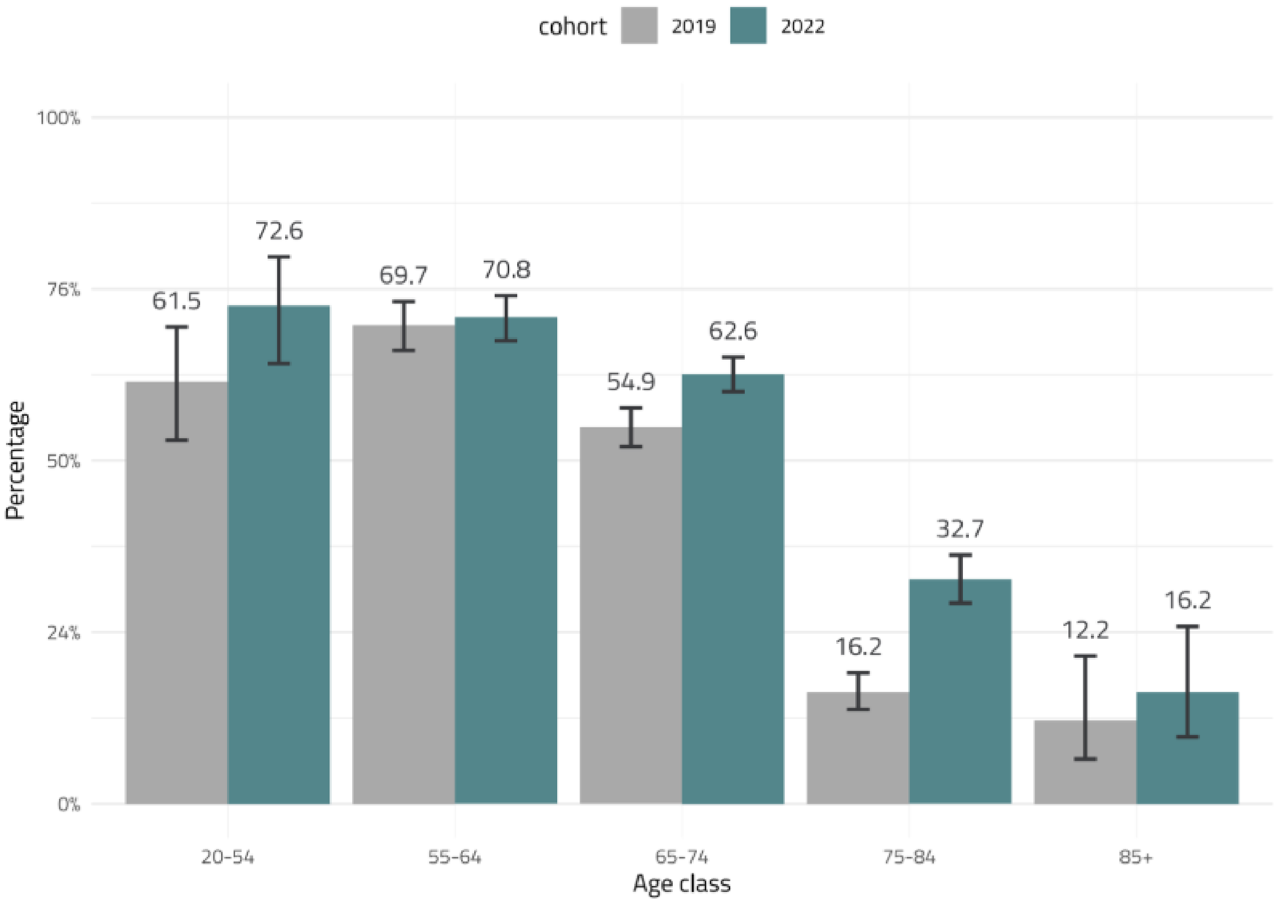

Figure S6. Radiotherapy within 365 days post-diagnosis, stratified by age class in the two cohorts.

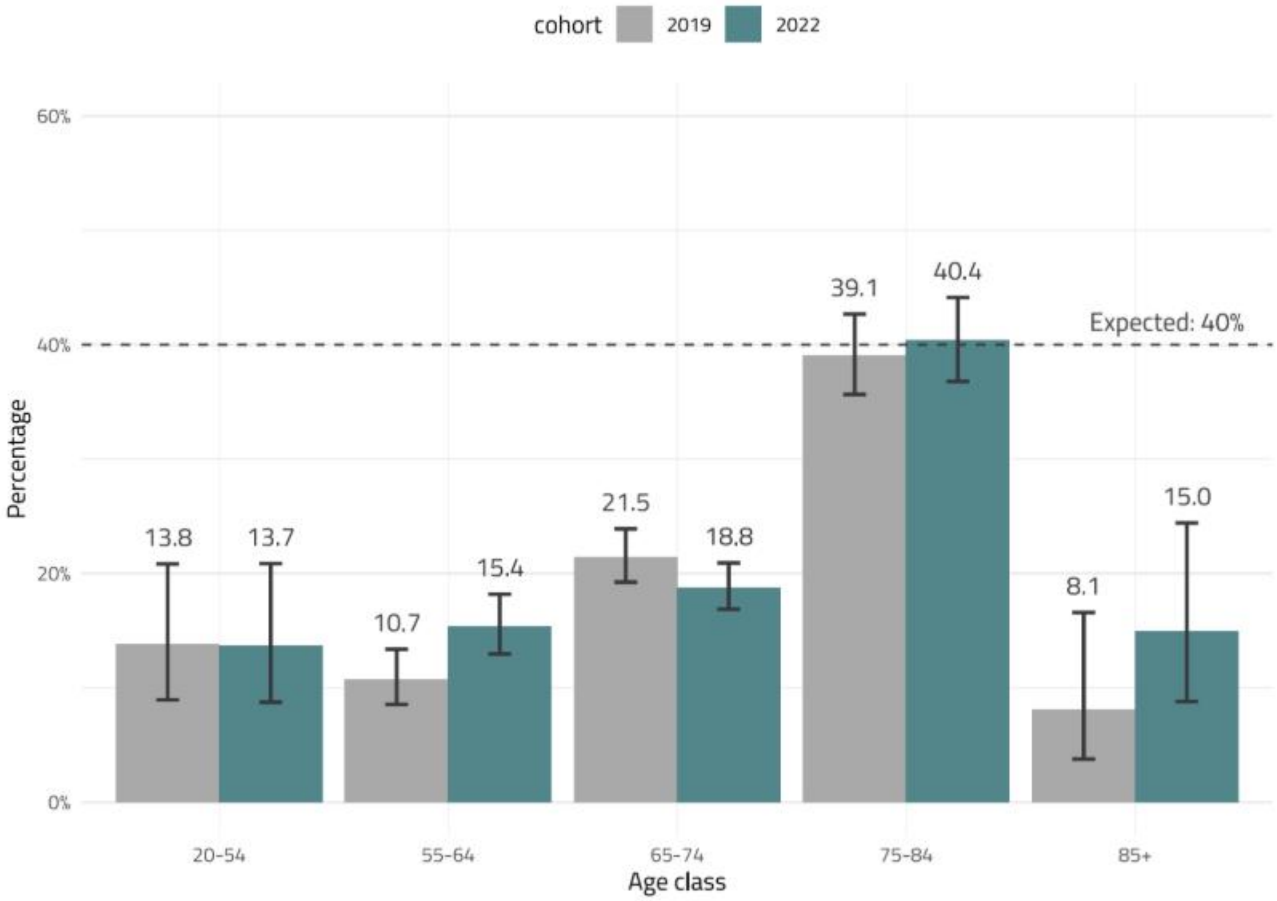

Supplement: Supplementary file 1 [file healthcare-14-01597-s001.zip › healthcare-4232604-supplementary.pdf]
